# Effects of Ranolazine and its Combination with Amiodarone on Rapid Pacing-induced Reentrant Atrial Tachycardia in Rabbits

**DOI:** 10.19102/icrm.2021.120304

**Published:** 2021-03-15

**Authors:** Isaac Aidonidis, Vassileios Simopoulos, Konstantina Dipla, Apostolia Hatziefthimiou, Rodopi Stamatiou, Ioannis Skoularigis, Paschalis-Adam Molyvdas

**Affiliations:** ^1^Department of Physiology, University of Thessaly, School of Medicine, Larissa, Greece; ^2^Department of Cardiac and Thoracic Surgery, University Hospital of Larissa, School of Medicine, University of Thessaly, Thessaly, Greece; ^3^Department of Sport Sciences at Serres, Aristotle University of Thessaloniki, Thessaloniki, Greece; ^4^Department of Cardiology, University Hospital of Larissa Medical School, University of Thessaly, Larissa, Greece

**Keywords:** Amiodarone, atrial pacing, atrial tachycardia, rabbit heart, ranolazine

## Abstract

Ranolazine (RAN) has previously been shown to lower the onset of cholinergic atrial fibrillation in intact animals; however, its efficacy in the setting of atrial tachycardia (AT) is unknown. The purpose of this study was to investigate the effects of RAN alone or in combination with amiodarone (AMIO) on rapid pacing-evoked right AT in rabbit hearts. Right atrial monophasic action potentials (MAPs) were recorded in 11 anesthetized rabbits, using combination MAP pacing catheters. Vulnerability to AT was tested by employing consecutive trains of rapid burst pacing prior to and after 2.4 mg/kg of RAN alone delivered intravenously and then in combination with 3 mg/kg of AMIO as a 15-minute infusion. Primary endpoints were postdrug AT reproducibility as well as cycle length (CL) and tachycardia duration. MAP duration at 75% repolarization and the effective refractory period (ERP) were assessed during programmed pacing to calculate the atrial postrepolarization refractoriness (aPRR = ERP – MAPD_75%_). AT was elicited in eight out of 11 rabbits; only these animals were included for further investigation. RAN did not abolish the inducibility of AT in any experiment; however, it prolonged its CL (baseline vs. RAN: 120 ± 16 ms vs. 138 ± 18 ms; p = 0.053). Supplemental AMIO further increased the AT CL (baseline vs. RAN + AMIO: 120 ± 16 ms vs. 152 ± 23 ms; p = 0.006), without affecting arrhythmia reinducibility. Slowing of the tachycardia after RAN or RAN + AMIO was associated with spontaneous termination of the arrhythmia. RAN prolonged the aPRR significantly, while AMIO in addition to RAN potentiated this effect. Neither RAN alone nor its combination with AMIO abolished the elicitation of AT in this model. However, both agents synergistically prolonged the aPRR, resulting in the slowing of AT and promoting spontaneous termination of the arrhythmia.

## Introduction

Atrial tachycardia (AT) frequently occurs under pathological conditions but may also be encountered in individuals that usually have no cardiac abnormalities. Increased automaticity or triggered activity and macro-reentry might contribute to its generation according to the last consensus for the classification of AT.^1^ More recently, ATs were observed secondary to catheter ablation for atrial fibrillation (AF) and characterized as more complex and symptomatic than AF itself.^2^ For these tachycardias, AF-like pharmacological management in the initial postablation period has been recommended using drugs belonging to Ic/III classes.^3^ However, as these agents can also affect ventricular electrophysiology, they may increase the risk of triggering ventricular proarrhythmia, limiting their use against AT, which per se is not a life-threatening arrhythmia.

Ranolazine (RAN) is a late sodium (Na^+^)-current (INaL) inhibitor at normal heart rates, acting almost equipotently in the atria and the ventricles at therapeutic doses. An atrial-selective peak Na^+^ current (INa)-blocking effect has been experimentally reported only at rapid activation rates,^4,5^ such as during tachypacing or an atrial tachyarrhythmia. Remaining trapped in the Na^+^ channel receptor, RAN impedes recovery from inactivation and results in a prolongation of refractoriness more than the action potential duration, which is labeled as postrepolarization refractoriness (PRR).^6–8^ Considering its rapid unbinding at the end of repolarization, the latter effect of RAN becomes strongly dependent on the duration of the diastolic interval. Consequently, RAN’s blocking effect on the INa channel further increases when the diastolic period becomes shorter or even is totally absent, which occurs, for example, during AF. Whether this mechanism might play a role in the effectiveness of RAN in suppressing AT-like cholinergic AF has not been investigated. Therefore, this study aimed to find out whether RAN, alone or in combination with amiodarone (AMIO), may be an alternative antiarrhythmic strategy for AT in structurally intact hearts.

## Methods

All applicable international, national, and/or institutional guidelines for the care and use of animals were followed. Experimental protocol procedures were approved by the institutional animal care committee. The study was conducted in accordance with the guidelines of the European Council on Animal Care.

### Surgical preparation

For this study, New Zealand white rabbits of either sex and weighing 3.0 to 3.5 kg were used. Following initial anesthesia with intramuscular ketamine hydrochloride (35 mg/kg) to accomplish sedation, routine preoperative interventions were performed; specifically, a peripheral vein catheter was inserted in the marginal ear vein (*Vena auricularis*), and subcutaneous needle electrodes for recording the surface electrocardiogram (ECG) were placed as required. Under continuous inspection of the ECG, lidocaine 2% was injected locally to facilitate painless preparation of the trachea; sufficient relaxation was accomplished with 0.02 to 0.15 mg/kg of intravenous pancuronium bromide immediately before proceeding with tracheostomy and intubation. After confirming the proper placement of the tube that allowed symmetric ventilation of both lungs, the animal was connected to a volume-controlled ventilator using room air mixed with oxygen (Harvard Apparatus, Holliston, MA, USA). The ventilator was set up with a 40 cycles/min respiratory rate and a stroke volume of 7 to 10 mL/kg. Next, to achieve deeper anesthesia, thiopental sodium (15–30 mg/kg) was slowly injected, enabling surgical exposition of both external jugular veins to allow the insertion of monophasic action potential (MAP) catheters. Body temperature was maintained between 38°C and 40°C during the entire experiment using an electrically heated pad.

### Recording system

Two recording/pacing MAP combination electrodes (Hugo Sachs Electronik, March-Hugstetten, Germany) were adequately soaked in saline before being introduced into the vein and advanced into the right atrium. When the tip electrode of the catheter came into soft contact with the atrial wall, a biphasic atrial signal was recorded and displayed on a multichannel monitor oscilloscope (RM-6000 polygraph amplifier system; Nihon Kohden, Shinjuku, Japan), equipped with a thermal array recorder (WS-682G; Nihon Kohden). Then, the pressure at this position was gently increased via gradual hardening of the distal part of the electrode until the pattern of the electrical signal became positive monophasic. Alterations of the MAP upstroke amplitude were frequent due to enhanced atrial free-wall compliance, but the repolarization time course remained stable, provided that the electrode was not displaced. The MAP signals were amplified by a high input impedance, MAP direct current–coupled, isolated, differential preamplifier (AB-601 G; Nihon Kohden) at a 5,000-Hz frequency. ECG and MAP recordings were manually evaluated.

### Pacing and arrhythmia-induction protocols

Programmed atrial stimulation was performed both prior to and after drug administration, using trains of seven consecutive basic drive stimuli at a 200-ms cycle length (CL) followed by a premature extrastimulus at 10-ms decrements until no atrial response was observed (Grass stimulator, model S8800; Grass Instrument Co., Quincy, MA, USA). Then, the extrastimulus CL was increased in 5-ms steps until a local atrial response was recorded. For pacing, a 2-ms pulse duration, twice the threshold voltage was applied. The atrial PRR (aPRR) was calculated following determination of the effective refractory period (ERP) and MAP duration at 75% repolarization measured during basic drive pacing. Following assessment of the aPRR, short burst pacing frequency series were applied to elicit sustained AT (sAT). Bursts consisting of five to seven rapid fixed-rate stimuli were repeated sequentially after programmed pauses of four seconds. Reproducibility of tachycardia was attempted at least twice to test whether the ATs resulted from different foci based on MAP morphology. Overdrive pacing (ODP) was performed to terminate incessant ATs, which were left to run for at least 60 seconds after their initiation. For changes in the CL of AT, diastolic intervals of three to five consecutive atrial MAPs were considered.

### Drugs

Ranolazine dihydrochloride (R6152) was purchased from Sigma-Aldrich (St. Louis, MO, USA).

### Electrophysiological determinations

For the purpose of this study, the following terms were defined as indicated:

- AT: A fast regular atrial rhythm reproduced by atrial burst pacing defined based on MAPs recorded from the right atrium and/or P-waves on the surface ECG
- Incessant AT: Each AT lasted over 60 seconds; these tachycardias were associated with hemodynamic deterioration and terminated using ODP
- sAT: Refers to an AT of at least 30 seconds or more than 100 atrial beats but lasting 60 seconds or less
- Nonsustained AT (nsAT): Refers to an AT of less than 30 seconds or more than six atrial beats
- ERP: Refers to the longest coupling interval between the last stimulus of the basic drive and single premature extrastimulus that failed to elicit a propagated atrial response
- aPRR: Refers to the difference between the ERP and the action-potential duration at 75% repolarization (ERP – MAPD_75%_).

### Statistics

Statistical analysis was performed using a one-way analysis of variance with repeated measures, followed by a Bonferroni adjustment. Analyses were performed with GraphPad Prism 5.01 (GraphPad Software, La Jolla, CA, USA). A p-value of less than 0.05 (or less than 0.016 following the Bonferroni adjustment) was considered to be statistically significant.

## Results

Among a total of 11 rabbits subjected to high-frequency stimulation, eight animals developed reproducible AT; the remaining three rabbits did not develop arrhythmias and were allowed to recover from the anesthesia. Control burst pacing elicited sAT, which required ODP for termination. The tachycardias developed and showed diastolic intervals and normal shaped P-waves on the surface ECG that differed from typical atrial flutter waves.

In contrast with control tachycardias, following the administration of RAN or RAN + AMIO, tachycardias were terminated spontaneously. However, these tachycardias still met the criteria of sustainability (≥ 30 seconds) **(Table 1)**. The number of pacing attempts to induce AT following drug administration was increased relative to baseline conditions. When the animals received RAN or the combination regimen, runs of nsAT were revealed more frequently before the tachycardia developed into a sustained form. However, sAT after RAN or RAN + AMIO administration converted much earlier to sinus rhythm without the need for ODP **(Table 1)**.

**Figure 1:**
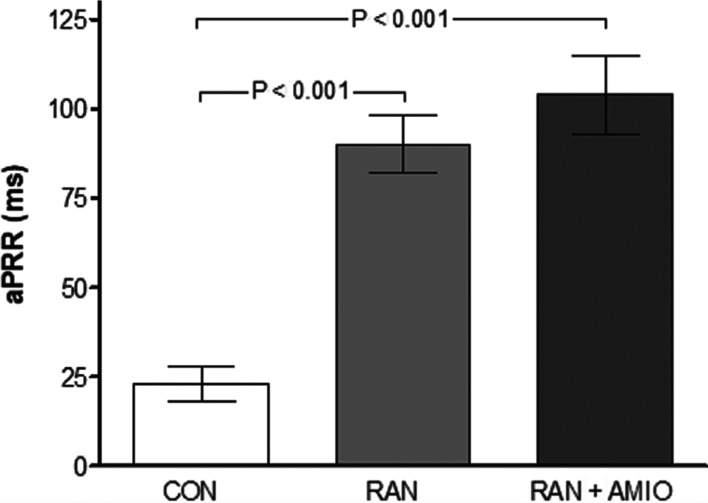
RAN significantly prolonged aPRR; the addition of AMIO tended to strengthen the RAN’s effect on refractoriness. CON: control; AMIO: amiodarone; aPRR: atrial post-repolarization refractoriness; RAN: ranolazine.

RAN administered after completing the control pacing protocol significantly decreased atrial conduction and prolonged the aPRR **(Figure 1)** as was reflected by the earlier appearance of 2:1 atrial block relative to with the control pacing **(Table 1)**. Atrioventricular (AV) conduction time was modestly prolonged with RAN, but this prolongation was potentiated after AMIO.

**Figure 2:**
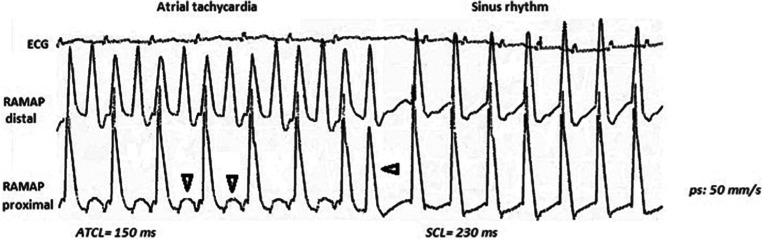
Mechanism possibly involved in the spontaneous termination of AT induced after RAN. MAPs recorded simultaneously from the right atrial endocardium during the course of tachycardia (left side of the figure) and after its termination (right side of the figure) show a 2:1 atrial block (vertical arrows) in the proximal atrial region, whilst the distal MAP electrode reveals a regular activation pattern. Elimination of block in the proximal area (horizontal arrow) was associated with conversion of the tachycardia into sinus rhythm. In the proximal area, phase 0 of MAP started earlier than in the distal phase in correspondence with the beginning of P-wave on the surface ECG. Note that only half of the atrial depolarizations during tachycardia reached the ventricles (AV block). AT: atrial tachycardia; AV; atrioventricular: ECG: electrocardiogram; MAP: monophasic action potentials; RAN: ranolazine.

Conduction block during AT was observed only after RAN in MAPs recorded from the right atrium, supporting the atrial-selective depressing effect of RAN on conduction at high activation rates **(Figure 2)**. This may partially explain the higher incidence of spontaneous conversion of ATs to sinus rhythm under RAN.

**Figure 3:**
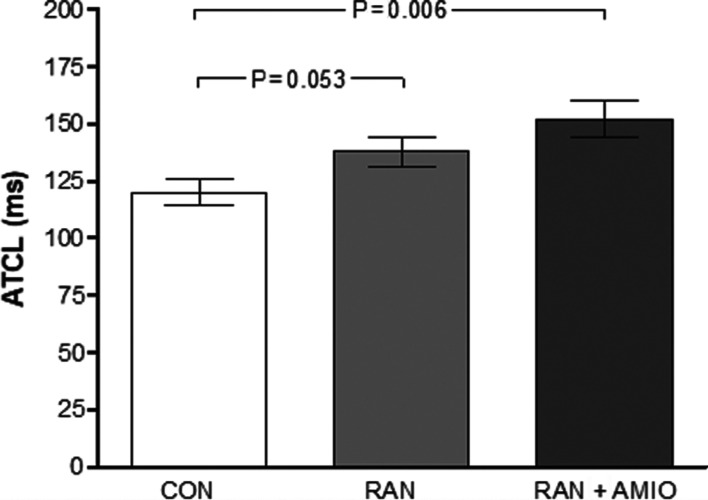
RAN alone yielded a better course of AT; the combination of RAN and AMIO significantly improved its efficacy by further increasing the AT CL. AT: atrial tachycardia; RAN: ranolazine; AMIO: amiodarone; AT CL: cycle length of the atrial tachycardia.

**Figure 4:**
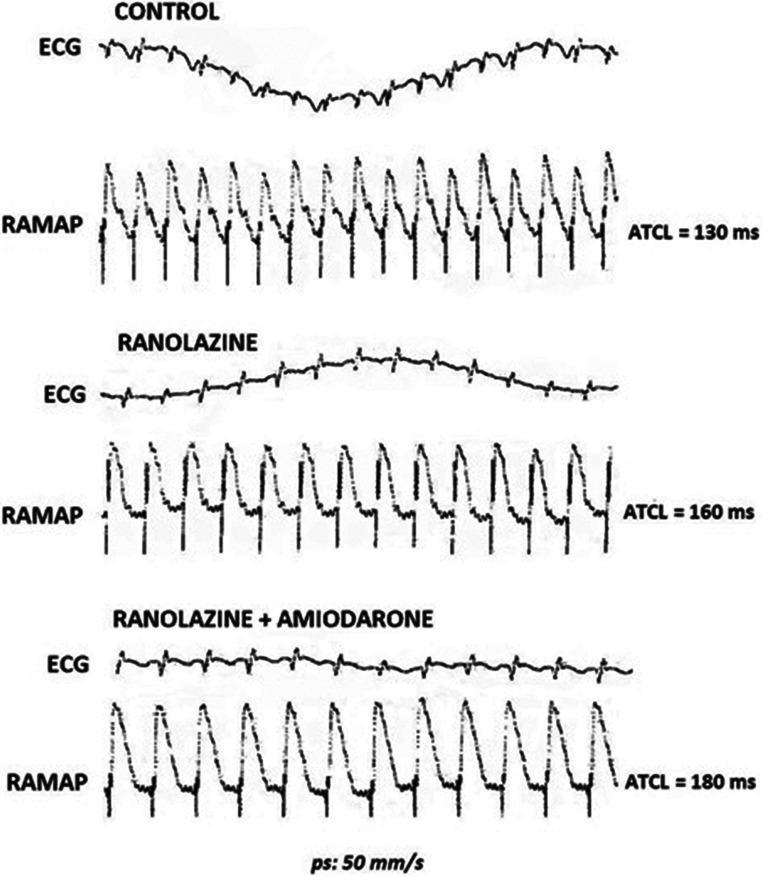
Effects of RAN or its combination with AMIO on pacing-induced sAT during control pacing conditions (baseline). Although the inducibility of the tachycardia was not abolished by RAN or RAN + AMIO, both treatment regimens effectively increased the AT CL. *ps*: paper speed. RAN: ranolazine; AMIO: amiodarone; AT CL: cycle length of atrial tachycardia; sAT: sustained atrial tachycardia.

In addition to spontaneous AT termination with RAN or RAN + AMIO, both medication schemes resulted in AT CL prolongation **(Figure 3)**. Greater AT CL lengthening was obtained after AMIO supplementation, suggesting the potentiation of aPRR prolongation rather than a reinforcement in conduction delay **(Table 1)**. A representative experiment is depicted in **Figure 4**, displaying the effects of RAN alone versus RAN + AMIO on the AT CL in a rabbit with reproducibly inducible tachycardias. Afterdepolarizations were not observed at the onset or the termination of AT in MAPs of the right atrium.

## Discussion

This study shows, for the first time, the effects of RAN alone or in combination with AMIO in a rapid pacing–elicited AT model. Neither RAN nor RAN + AMIO abolished the inducibility of sAT in any of the experiments; however, they promoted spontaneous conversion of tachycardia to sinus rhythm and significantly prolonged its CL. These effects are possibly attributed to RAN’s capacity to markedly increase aPRR and the intra-atrial conduction time, as indicated by the 2:1 atrial MAP blockade observed at longer pacing coupling intervals (ie, intra-atrial block). The addition of AMIO only moderately reinforced RAN’s effect on aPRR, while it left atrial conduction unaffected as compared with as seen when using RAN alone.

The inefficacy of RAN to completely suppress the inducibility of sAT could be associated with the relatively slower rates of tachycardia relative to atrial flutter or fibrillation.^9^ This may point out the importance of the diastolic interval duration for RAN’s efficiency in abolishing this arrhythmia. RAN needs high activation rates to depress atrial INa and, thus, to block areas of delayed conduction.^10,11^ However, when enhanced abnormal automaticity is predominantly based on calcium (Ca^++^)-induced triggered activity, such as delayed afterdepolarizations, RAN may prevent ectopic-beat formation and arrhythmogenesis by the inhibition of INaL at normal heart rates.

At normal sinus rhythm rates, RAN is least likely to block areas with delayed conduction so that silent reentry can persist and be activated under certain conditions. Interestingly, Burashnikov et al.^12^ showed that prolongation of atrial repolarization with the atrial-selective IKr inhibitor E-4031 potentiated RAN’s INa-blocking effect in the canine atria possibly by way of longer binding to the Na^+^ channel receptor.

The fact that acute AMIO administered in addition to RAN was found to further increase aPRR but not yield better prevention of AT inducibility may support efforts to hypothesize about a prominent role of conduction over refractoriness. In our experiments, a gradual aPRR prolongation post-AMIO was well correlated with lengthening of the AT CL rather than AT suppression. Furthermore, intermittent blockade of atrial excitability detected in intra-atrial MAPs during tachycardia was contemplated only after RAN and associated with shorter duration and spontaneous reversion of AT to sinus rhythm. We assume that conduction block in the vicinity of reentry may be partly responsible for the spontaneous cessation of AT when the reentrant impulse falls onto a nonexcitable region. This might also explain why more burst pacing attempts were necessary to reproduce sAT after RAN as compared with under baseline conditions.

Drugs used against extranodal ATs mostly belong to the Ic or III classes and are atrial-nonselective INa blockers that occasionally bear the risk of ventricular proarrhythmia. In comparison, RAN and AMIO in combination with one another have been safely applied in patients with postoperative AF in the setting of cardiac surgery.^13^ Our experimental data show that RAN, an anti-ischemic drug, converted sAT to nsAT in about half of the animals and decreased the rate and the duration of reproducibly inducible sAT.

It is reasonable to assume that RAN may exert its antiarrhythmic action by prolonging intra-reentry and peri-reentry conduction velocity and refractoriness; however, the strength of this action is rate- and possibly dose-dependent.^14^ Accordingly, the increased propagation time of high-rate atrial depolarizations after RAN support a conduction-depressing effect of this compound in rapidly depolarizing atria.

### Study limitations

This was a very small study in which only eight of the 11 included animals developed reproducible ATs and there is a need for larger human studies to confirm its clinical relevance. Furthermore, plasma levels of both agents were not measured to determine dose-dependent effects of RAN and AMIO on AT. On the other hand, higher doses of these agents could not be used due to reductions in contractility and AV conduction, resulting in cardiac arrest. Another limitation was the lack of a precisely defined location of the atrial MAP catheters within the right atrium. Proximal or distal atrial MAPs were characterized merely upon a time-related manner by superimposing the starting point of phase 0 from the beginning of the P-wave on the ECG. Collectively, although these experimental data cannot be directly extrapolated to human subjects with structural heart disease, their synergistic electrophysiologic actions support the contention that a combination of RAN and AMIO for the termination and prevention of sAT might be better than either of these agents alone.

### Clinical implications

Although AT is a relatively rare form of supraventricular tachyarrhythmia, it has not historically been considered life-threatening in individuals without structural heart disease. However, class Ic/III agents indicated for this arrhythmia may accentuate the risk of ventricular proarrhythmia, especially in patients with ischemic heart disease largely due to their atrial-nonselective action. Considering the effectiveness of the RAN–AMIO combination strategy and the lower incidence of proarrhythmia in experimental and clinical settings, the present findings argue for the need for an alternative treatment even in patients with a history of coronary heart disease and concomitant AT. Moreover, chronic administration of AMIO with RAN^15,16^ might be expected to result in a stronger antiarrhythmic efficacy.

## Conclusion

RAN possesses antiarrhythmic efficacy against AT elicited by rapid burst stimulation in anesthetized rabbits. Although RAN alone or in combination with AMIO did not effectively suppress AT reproducibility in this experimental model, both drugs showed acute synergistic electrophysiologic action in prolonging aPRR but not atrial conduction. The shorter duration and spontaneous termination of sAT after RAN were possibly related to increased refractoriness and RAN’s local conduction block during the course of tachycardia, with the latter in line with in vitro findings demonstrating a depressive effect of RAN on atrial conduction only at high activation rates.

## Figures and Tables

**Table 1:** Basic Electrophysiologic Variables and AT Inducibility-termination Techniques During Control Pacing and After Drug Administration

	2:1 AVB (ms)	2:1 IAB (ms)	AT Duration (s)	Burst Attempts for AT Induction	Spontaneous Termination of AT	Type of AT
CON (n = 8)	110 ± 9	90 ± 5	0 80 ± 11	3 ± 1	0/8	Incessant
RAN (n = 8)	120 ± 11 (p = 0.066)	100±5 (p < 0.01)	40 ± 5	10±3	5/8	nsAT sAT
RAN + AMIO (n = 8)	150 ± 14 (p < 0.001)	100 ± 4 (p < 0.001)	37 ± 3	13±3	7/8	nsAT sAT

AMIO: amiodarone; AT: atrial tachycardia; AVB: atrioventricular block; CON: control pacing; IAB: intra-atrial block; n: number of animals; nsAT: nonsustained AT; ODP: overdrive pacing; RAN: ranolazine; sAT: sustained AT.

Data are presented as mean ± standard deviation; p-values indicate RAN or RAN + AMIO vs. CON.
